# Psychiatrist-led hepatitis C (HCV) treatment at an opioid agonist treatment clinic in Stockholm– a model to enhance the HCV continuum of care

**DOI:** 10.1186/s12888-025-06733-3

**Published:** 2025-03-27

**Authors:** Per-Erik Klasa, Mikael Sandell, Soo Aleman, Martin Kåberg

**Affiliations:** 1 Prima Maria OAT Clinic, Stockholm, Sweden; 2 Department of Infectious Diseases, Karolinska University Hospital, Stockholm, Sweden; 3 Department of Medicine Huddinge, Infectious Diseases, Karolinska Institutet, Stockholm, Sweden; 4 Department of Global Public Health, Karolinska Institutet, Sprututbytet, S:t Görans sjukhus, Akutvägen 29, Stockholm, 112 81 Sweden; 5 Stockholm Needle Exchange, Stockholm Centre for Dependency Disorders, Stockholm, Sweden

**Keywords:** HCV treatment, OAT, Continuum of care, Task shifting

## Abstract

**Background:**

People with opioid agonist therapy (OAT) represent a population with an increased hepatitis C (HCV) prevalence. Recent studies provide strong evidence regarding effective HCV treatment outcomes and low levels of reinfection in this population. Increased access to HCV care for people with OAT is essential to reach the WHO goal of eliminating HCV as a major public health threat by 2030.

**Methods:**

The Maria OAT clinic, located in central Stockholm, provides OAT for approximately 500 patients. The majority have a history of injection drug use. In October 2017, psychiatrist-led HCV treatment was initiated, with remote consultation support from the local infectious diseases clinic. All OAT staff participated in HCV-specific education to increase HCV awareness. To evaluate HCV treatment outcomes for this model of care, we examined sustained virological response (SVR) and reinfection rates between January 2018 and December 2022.

**Results:**

Between October 2017 and June 2022, 133 participants received HCV treatment through weekly administrations or directly observed treatment. 72% were men, and the overall mean age was 44.7 years. Six participants were retreated, giving a total of 139 treatment initiations. All were HCV RNA negative at end of treatment, and 88% reached SVR. A total of 11 reinfections post SVR were noted, with a reinfection rate of 7.3/100 person-years (95% CI 4.1–12.9).

**Conclusion:**

Overall, successful HCV treatment results and levels of reinfections consistent with the literature were achieved. Bringing HCV diagnostics and treatment to an OAT clinic constitutes a good example of enhancing the HCV continuum of care. Furthermore, HCV treatment education for psychiatrists, addiction specialists and staff at OAT clinics makes HCV care more sustainable, as specifically noted during the COVID-19 pandemic. This successful model of care, introducing HCV treatment by psychiatrists on-site at OAT clinics, has now been further implemented at other OAT clinics in Stockholm.

## Background

An estimated 50 million people worldwide are infected with hepatitis C virus (HCV) [1]. People who inject drugs (PWID) and people with opioid agonist therapy (OAT) are often overlapping populations with an increased prevalence of hepatitis C virus (HCV) infections. Recent studies provide strong evidence regarding the effectiveness of HCV treatment with direct-acting antivirals (DAAs) and low levels of reinfections among these populations [2–6]. Increased access to HCV care for people with OAT is essential to reach the WHO goal of eliminating HCV as a major public health threat by 2030. The 2016 WHO HCV elimination targets include an 80% reduction in new cases and a 65% reduction in HCV-related deaths [7, 8]. In 2021, new targets were introduced and defined as an absolute annual HCV incidenceof <5/100,000 in all persons and <2/100 in PWID [9, 10].

Although access to OAT has increased in Sweden over the past decade, national coverage remains low compared to other European Union countries, with only 62 OAT recipients per 100,000 inhabitants, compared to the EU average of 100 per 100,000 [11]. OAT reduces the risk of HCV among PWID, and its effectiveness is further enhanced when combined with needle and syringe programs (NSP) [12]. In a systematic review from 2023, OAT coverage among PWID in Sweden was reported in the higher interval of > 40 OAT recipients per 100 PWID, while NSP coverage was at a moderate level of 100 to 200 needle and syringes distributed per PWID annually [13].

Since 2018, HCV treatment in Sweden has been universal and fully reimbursed. However, DAAs must be prescribed by, or in consultation with, a physician at an infectious diseases (ID) or gastroenterology clinic with experience in treating patients with HCV [14, 15]. Furthermore, HCV treatments must be registered in the national quality register InfCare Hepatitis to ensure national follow-up and quality assurance of care.

Even with universal access to HCV treatment, patients still need to be linked to care. As numerous studies have shown, there are multiple factors that might negatively affect the ‘HCV care cascade’ or the ‘continuum of care’ defined as retention in every step from diagnosis to reaching cure [16–18]. Over time, from screening of anti-HCV, through confirmatory HCV RNA, linkage to a specialist assessment, follow-up visit for fibrosis assessment and treatment start, a great proportion of patients might be lost to follow-up (LTFU) [19]. Barriers to the HCV care cascade might include invasive testing methods, limited access to pangenotypic treatments, and logistical challenges like travel costs and clinic distances. Systemic factors such as inadequate funding, stigma, and weak governance can further hinder engagement, while personal factors like low motivation might be addressed through peer-support programs [18, 20]. By addressing these factors and treating people with HCV geographically closer to where they already access services, aiming for a ‘one-stop-shop’, the continuum of care could be improved [17]. Hence, HCV treatment should be offered in settings such as substance use clinics, OAT clinics, prisons and at needle and syringe programs (NSP).

Before the introduction of DAA, overall lifetime HCV treatment uptake among OAT participants in Sweden was low, with only 1–6% treated [21–23]. An observational study with data from the Swedish Prescribed Drug Registers noted an estimated cumulative DAA treatment uptake of 28% among OAT participants between 2014 and 2017 [24]. However, these estimates represent a time when restrictions regarding level of fibrosis was still present in the Swedish HCV treatment guidelines.

In 2019, there were an estimated 29 700 viremic HCV infections in Sweden, and an HCV transmission modelling study from 2021 concluded that Sweden would achieve and exceed the WHO targets for diagnosis, treatment and liver-related death by 2030. However, fully achieving all WHO targets, including a substantially reduced incidence, would require expanding harm reduction programs (including OAT) to engage and treat more than 90% of PWID with HCV in these programs [25].

Since the introduction of DAA, several innovative models of HCV care targeting OAT participants and PWID have emerged. These include decentralized mobile clinics with point-of-care testing and treatment, peer-led test-and-treat programs, and community pop-up clinics to simplify access to services, along with the integration of HCV treatment directly into OAT settings [26–30]. However, different countries, regions and setting have different challenges related to local guidelines and resources.

The Swedish national HCV elimination plan was published in July 2022. The elimination plan highlights that “a close collaboration with substance use disorder clinics is important” and that “treatment of HCV, in collaboration with an infectious disease clinic, should be offered directly at OAT clinics with diagnostics, investigation, treatment and follow-up on-site” [31].

In this study, we aim to methodologically describe how the first psychiatrist-led HCV treatment model of care was introduced at an OAT clinic in Stockholm, Sweden. Furthermore, we aim to evaluate the outcome of this model of care by assessing HCV treatment success, defined as sustained virological response (SVR) with a negative HCV RNA test 12 weeks after end of treatment (EOT), as well as the rate of reinfection during follow-up.

## Methods

### Setting and patients

In Sweden, OAT is managed only by specialist in psychiatry +/- specialists in addiction medicine (which is a sub-specialty of psychiatry). The Maria OAT clinic is located in central Stockholm and provides OAT (methadone, buprenorphine and buprenorphine/naloxone) for approximately 500 patients. Of these, 36% dose daily at the clinic, 36% have take-home doses from the clinic and 28% have fully prescribed OAT through the pharmacy, corresponding to the differentiated treatment groups defined below. The majority (82%) have a history of injecting drug use (IDU), and the primary injected opioid in Stockholm is heroin. Previous studies have noted a 69% viremic HCV prevalence among OAT patients in Sweden and that HCV has been poorly diagnosed and followed-up at OAT clinics [22, 32]. An unpublished report from the Maria OAT clinic noted that among all OAT patients in 2018 (*n* = 418), almost half (46%) were not tested for HCV or had an unknown HCV status in the digital medical chart. Of those tested (*n* = 225), 64% had a chronic infection, 25% had a cleared infection (spontaneously or treatment induced) and 11% had never been exposed to HCV (personal communication Tobias Nordin, Maria OAT clinic).

### Differentiation of OAT patients

The Maria OAT clinic offers a specialized OAT treatment approach, categorizing patients into three groups based on their treatment needs, as outlined below:

1. High treatment needs (HTN)– patients with clinical complexity and concomitant high-risk drug use, including IDU. The treatment focus has a pronounced harm-reduction approach with OAT medicine dispensed with daily monitoring.
2. Moderate treatment needs (MTN)– patients with clinical complexity to some degree but with treatment needs adequately addressed and less frequent drug-related high-risk presentation. Treatment is focused on rehabilitation with regular monitoring but with increased treatment autonomy.
3. Low treatment needs (LTN)– patients with minimal high-risk or problematic substance use and with stable medical, psychiatric and psychosocial conditions. Patients receive OAT medication through prescription and manage the OAT treatment on their own without supervised intake.

As part of the HCV model-of-care introduced in this study, all OAT patients at the Maria OAT clinic could access HCV testing and treatment on-site. However, most patients in the low treatment needs group and many patients in the moderate treatment needs group would also access HCV treatment through standard-of-care referrals to regional ID clinics.

### Introducing psychiatrist-led HCV treatment

In October 2017, psychiatrist-led HCV treatment (with consultation support from the local infectious diseases clinic) was initiated at the Maria OAT clinic. Prior to this, there were no HCV treatments offered on-site. Instead, OAT patients were referred to the specialized ID clinics, often resulting in a missed visit, particularly among OAT patients in the high treatment needs group (personal communication Per-Erik Klasa, Maria OAT clinic).

During 2017–2019, the Maria OAT clinic collaborated with the adjacent Stockholm NSP and performed liver stiffness measurements (LSM) with Fibroscan at the OAT clinic for those with viremic HCV. On a parallel level, an educational effort was initiated with the aim of increasing HCV knowledge among OAT staff, with a specific focus on teaching a psychiatrist at the clinic to manage and treat HCV on-site. This educational program was initiated by an ID specialist from the Karolinska University Hospital in Stockholm. In early 2020 the Maria OAT clinic’s staff also attended the course “Hepatitis C in Primary Care and Drug and Alcohol Settings Education Program” that specifically targeted Swedish psychiatrists and primary care physicians [33]. The course was developed by the Australasian Society for HIV, Viral Hepatitis and Sexual Health Medicine (ASHM) in collaboration with the Kirby Institute, UNSW Sydney and the International Network on Health and Hepatitis in Substance Users (INHSU). An evaluation of the course noted that “self-efficacy related to HCV management and treatment improved immediately following the delivery of this HCV educational program” [34].

**Fig. 1:**
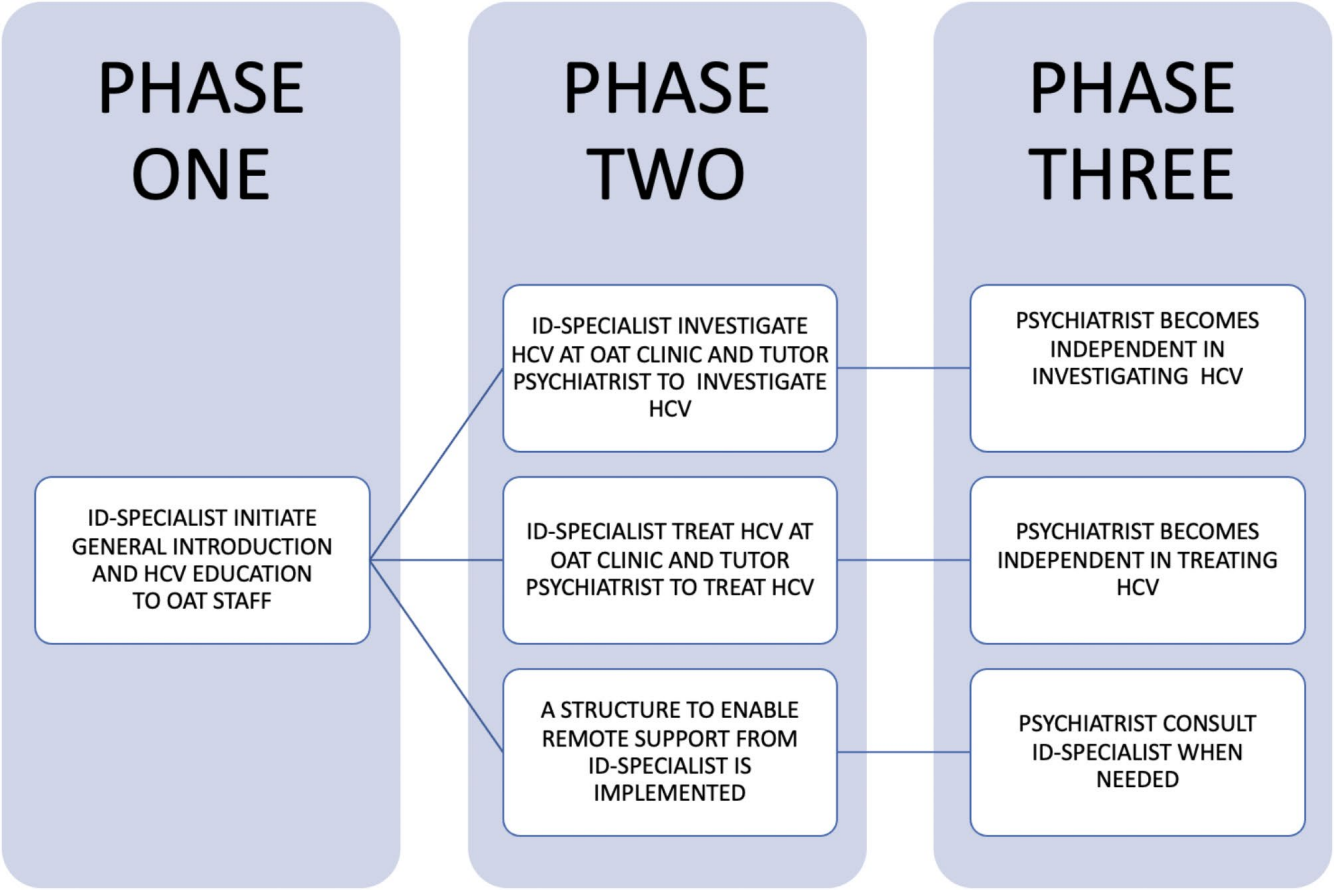
Process to initiate psychiatrist-led HCV treatment. ID = Infectious diseases, HCV = Hepatitis C

The overall HCV education concept at the Maria OAT clinic was to teach psychiatrists to be independent in HCV investigation, treatment and follow-up (Fig. 1). Briefly, during phase one the primary focus was to introduce treatment on-site and educate OAT staff on HCV. During phase two, hands-on clinical guidance to nurses and psychiatrists was provided by an ID specialist, twice a week. At this stage, the ‘remote consultation form’ for HCV treatment initiation was introduced for future consultations with an ID physician experienced in HCV treatment [35]. The form was initially developed by the Gastroenterological Society of Australia– Australian Liver Association and then adopted to current Swedish HCV treatment guidelines. The form contained information on patient data (name and date of birth), HCV history, prior HCV treatments, intercurrent medical conditions, current medication (checked for DAA interactions through University of Liverpool’s ‘HEP Drug Interactions’ [36]), laboratory results, liver fibrosis assessment (LSM with Fibroscan and/or APRI score) and a suggested choice of DAA treatment based on HCV genotype. Finally, in phase three, all investigations and treatments were performed by the OAT psychiatrists. At this point, there was no ID specialist on-site at the OAT clinic but a possibility to contact one on demand or more planned through a biweekly telemedicine HCV conference. The ‘remote consultation form’ was used as the basis for the HCV treatment consultations.

### HCV treatment

Participants were included for HCV treatment between 27th October 2017 to 17th June 2022.

During the first year, HCV treatment was initiated by the ID specialist, or by the psychiatrist with support from the ID-specialist on-site. From 2019, all treatments were initiated by the psychiatrist with remote ID specialist consultations when needed. All participants were assessed regarding level of fibrosis (F0-F4), most often with Fibroscan. Severe fibrosis could also be excluded using the algorithm APRI score < 1 in combination with age < 35 years and duration of IDU < 15 years [37]. Participants with cirrhosis (F4), i.e. an LSM > 12.5 kPa [38], underwent ultrasound prior to treatment initiation to rule out hepatocellular carcinoma and were planned for repeated ultrasound follow-ups post SVR, in accordance with Swedish HCV guidelines. Swedish guidelines allow for treatment of acute HCV as well as unlimited retreatments. The guidelines still demand HCV genotype testing before treatment start.

In this study, all HCV testing was performed through venipuncture and there was no access to capillary point-of-care tests such as dried blood spot or on-site HCV RNA testing. However, to facilitate HCV testing in this model of care, the Maria OAT clinic implemented HCV testing on-site to reduce the risk of missed diagnosis and LTFU. All laboratories in Stockholm that conduct HCV testing offer HCV RNA reflex testing for HCV-antibody positive samples to determine current HCV status.

All HCV-treated participants were HCV RNA tested at EOT, at SVR and then followed with repeated HCV RNA tests to the last negative HCV RNA test or a subsequent reinfection until December 31st, 2022.

### InfCare hepatitis

All HCV treatment data were registered in the national quality register InfCare Hepatitis [39]. The registry contains demographic data (age and gender) and HCV treatment data (fibrosis evaluation, blood tests including HCV serology/virology, HCV genotypes and prescribed DAA treatment). All HCV RNA follow-up tests post SVR were registered in InfCare Hepatitis.

### Statistics

Demographic data are presented as proportions, means or medians with ranges. All participants were followed with repeated HCV tests post treatment with SVR to identify possible reinfection. The actuarial method was used to define the time to reinfection as the midpoint between the last HCV RNA negative test and the following positive HCV RNA test. Reinfection rates were defined as the number of reinfections (n = x) per 100 person-years (x/100 PY), with 95% confidence intervals (CIs). Data were analysed using JMP^®^, Version 15, SAS Institute Inc., Cary, NC.

## Results

By June 2022, 133 participants had initiated HCV treatment on-site at the Maria OAT clinic. Six participants were retreated, giving a total of 139 treatment initiations. Thirty-five participants (25%) initiated HCV treatment through the ID-specialist together with the psychiatrist in phase two during the first year, while 104 participants (75%) were managed by the psychiatrist alone in phase three (Fig. 1). HCV treatment was mostly provided through directly observed treatment (DOT) or weekly administrations, with a minority initiating HCV treatment while in prison (*n* = 1) or in treatment homes (*n* = 13).

The demographics of treated participants and treatment groups are depicted in Table 1. Most treatments were started in the high treatment needs group (57,6%) and in the moderate treatment needs group (34,5%). Overall, 72% were men and mean age was 44.7 years (range 22–65). The distribution of genotypes (GT) was 49%, 41% and 10% for GT 3, GT 1 and GT 2, respectively. The majority had absent or mild fibrosis (63%), while 5% had LSM indicating liver cirrhosis. The treatment strategy was following Swedish HCV treatment guidelines, and the two most used DAA-treatment strategies were eight weeks of sofosbuvir/ledipasvir for GT 1 (25.2%) and 12 weeks of sofosbuvir/ledipasvir for GT 2 and 3 (44.6%).

**Table 1:** All HCV treatment initiations (*n* = 139)

	All HCV treatments (*n* = 139)	High Treatment Needs (HTN) (*n* = 80)	Moderate Treatment Needs (MTN) (*n* = 48)	Low Treatment Needs (LTN) (*n* = 11)
	n (%)	n (%)	n (%)	n (%)
Gender				
Men	100 (71.9)	59 (73.8)	35 (72.9)	6 (54.5)
Women	39 (28.1)	21 (26.3)	13 (27.1)	5 (45.5)
Age, years				
Mean (SD)	44.7 (10.4)	45.0 (9.3)	43.9 (11.5)	43.5 (11.9)
Median (range)	45 (22–66)	45 (25–63)	45 (22–65)	45 (28–59)
Genotype				
1	57 (41.0)	35 (43.8)	17 (35.4)	5 (45.5)
2	14 (10.1)	7 (8.8)	7 (14.6)	-
3	68 (48.9)	38 (47.5)	24 (50.0)	6 (54.5)
Fibroscan (kPa)	*n* = 124	*n* = 73	*n* = 42	*n* = 9
Mean (SD)	7.7 (6.5)	7.7 (6.7)	7.9 (6.9)	6.0 (1.2)
Median (range)	6.3 (2.9–59.3)	6.1 (2.9–59.3)	6.6 (3.2–49.7)	6.3 (3.9–7.4)
Fibrosis level	*n* = 129	*n* = 77	*n* = 43	*n* = 9
F0-F1	81 (62.8)	49 (63.6)	25 (58.1)	7 (77.8)
F2	26 (20.2)	12 (15.6)	12 (27.9)	2 (22.2)
F3	16 (12.4)	11 (14.3)	5 (11.6)	-
F4	6 (4.7)	5 (6.5)	1 (2.3)	-
Treatment				
GLE/PIB	22 (15.8)	12 (15.0)	9 (18.8)	1 (9.1)
GRZ/ELB	18 (13.0)	13 (16.3)	2 (4.2)	3 (27.3)
LED/SOF	35 (25.2)	19 (23.8)	14 (29.2)	2 (18.2)
SOF/VEL	62 (44.6)	35 (43.8)	22 (45.8)	5 (45.5)
SOF/VEL/VOX	2 (1.4)	1 (1.3)	1 (2.1)	-

* Retreated patients (*n* = 6): men 100%; mean age 48.7; GT 1 (*n* = 3), GT 3 (*n* = 3), fibrosis level F0-F1 (*n* = 3), F2 (*n* = 2), F3 (*n* = 1). HCV = Hepatitis C, SD = Standard deviation, F = Fibrosis level, GLE = glecaprevir, PIB = pibrentasvir, GRZ = grazoprevir, ELB = elbasvir, LED = ledipasvir, SOF = sofosbuvir, VEL = velpatasvir, VOX = voxilaprevir

**Fig. 2:**
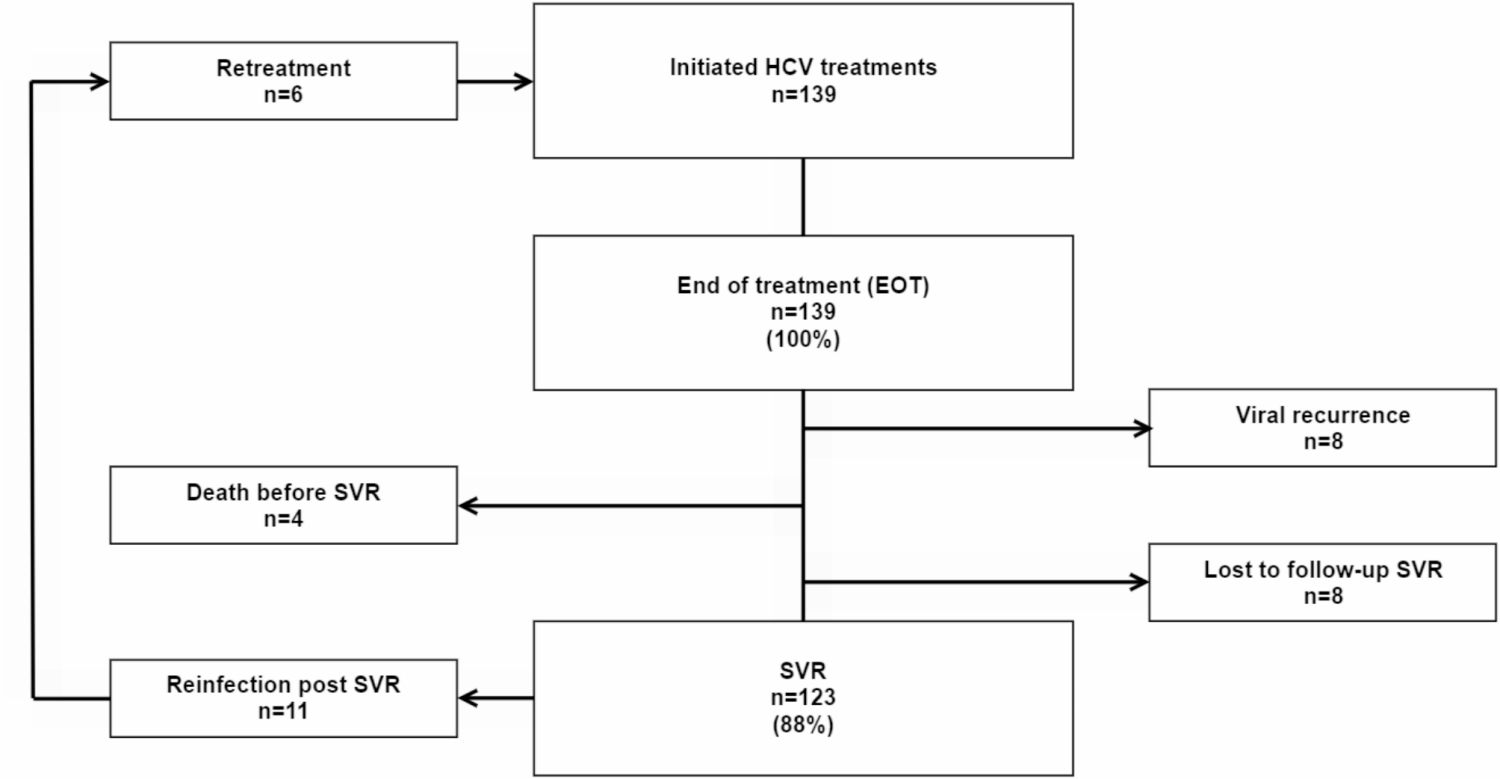
Flowchart of HCV-treated patients. HCV = Hepatitis C, EOT = End of treatment, SVR = Sustained virological response

All treated patients, 139/139 (100%), were HCV RNA negative at EOT and 123/139 (88%) reached SVR, with 8 viral recurrences, 4 LTFU and 4 deaths between EOT and SVR (Fig. 2). All viral recurrences between EOT and SVR were found in the high treatment needs group, while LTFU and deaths were found in both the high treatment needs group (LTFU *n* = 2, deaths *n* = 3) and in the moderate treatment needs group (LTFU *n* = 2, deaths *n* = 1). There were no demographic differences between the three OAT treatment groups regarding age and gender.

A total of 11 reinfections were noted post SVR (Fig. 2), over 151 person-years of follow-up, giving a reinfection rate of 7.3/100 PY (95% CI 4.1, 12.9). Most reinfections post SVR, 91% (10/11), occurred in the high treatment needs group, with a reinfection rate of 12.5/100 PY (95% CI 7.0-22.3), over 80 person-years follow-up. One reinfection noted in the moderate treatment needs group represented a reinfection rate of 1.5/100 PY (95% CI 0.2–10.9), over 64 person-years of follow-up. There were no reinfections in the low treatment needs group (6 person-years of follow-up).

Over the study period, the numbers of HCV treatment initiations were: 2017, from October (*n* = 5); 2018 (*n* = 35), 2019 (*n* = 38); 2020 (*n* = 22); 2021 (*n* = 27) and in 2022, until September (*n* = 12).

## Discussion

In this study, we successfully introduced psychiatrist-led HCV treatment at an OAT clinic in Stockholm. Overall, we noted great HCV treatment results and levels of reinfections consistent with the literature.

In a review from 2018, Hajarizadeh et al. concluded that treatment completion was 97.4% and 96.9% and SVR was 90.7% and 87.4% among those with OAT and those with recent IDU, respectively [3]. The review also concluded that people with recent IDU had a generally lower level of SVR than those with no history of IDU and that LTFU at SVR was the main contributor rather than virological failure. These data correspond well to our data where treatment completion was 100% and SVR was 88%. However, the decreased level of SVR in our data reflect both LTFU and viral recurrence between EOT and SVR.

A meta-analysis from 2020 investigating HCV reinfection after successful antiviral treatment among PWID noted a reinfection rate of 6.2/100 PY among people with recent IDU and 3.8/100 PY among those receiving OAT [6]. In further stratified analyses among people with OAT, reinfection rates were 1.4/100 PY and 5.9/100 PY among those with no recent drug and those with recent drug use, respectively. In adjusted rate ratio analyses, those with recent drug use were at a 3.5-fold higher risk of reinfection, than those with no recent drug use [6]. The overall reinfection rate at the Maria OAT clinic was, 7.3/100 PY, but was higher in the high treatment needs group (12.5/100 PY), indicating a higher level of high-risk drug use. Although the pooled reinfection rates in the meta-analysis were lower than those in our study, the meta-analysis included studies with reinfection rates between 16.7 and 23.8/100 PY among DAA-treated PWID and persons with OAT [40, 41].

Another international multicenter study prospectively followed HCV treated OAT participants for reinfection up to 3 years after successful DAA treatment. The overall reinfection rate was 1.7/100 PY and 1.9/100 PY among those with recent drug use. However, only 20% reported IDU during follow-up and the authors concluded that reinfection rates could be underestimated as the study participants may have represented a group with higher stability and lower risk for reinfection. Additionally, the high level of LTFU may have represented participants with lower stability, underestimating reinfection rates [42].

In a study from Canada (*n* = 482) with 46% receiving OAT, 91% of reinfections occurred among people with known recent IDU at the time of treatment start, which corresponds well to our data. The overall reinfection rate was 3.6/100 PY and among those with recent IDU, the reinfection rate was 6.6/100 PY. In a study from the USA (*n* = 141), overall low levels of reinfections among people receiving OAT were noted (1.1/100 PY), but were higher among those with recent IDU, 7.7/100 PY [43].

A study investigating HCV treatment at the Stockholm NSP, noted that PWID with OAT and concurrent IDU, attending the NSP, received HCV treatment to a higher extent in settings other than the NSP, mainly in OAT clinics and ID clinics. However, the reinfection rates did not differ between those treated at the NSP, 8.4/100 PY (95% CI 5.3, 13.2) or at other clinics, 9.9/100 PY (95% CI 6.9, 14.3) [44].

Altogether, as noted in a modelling study, persistent high HCV treatment rates in PWID will result in an initial increased number of reinfections that will be curbed over time [16]. Thus, reinfections will occur in high-risk populations, but retreatment needs to be recognized as a vital part of an effective HCV elimination strategy [45]. Given that Sweden generally has low OAT and NSP coverage, increased access to such harm reduction interventions need to be a future priority as pinpointed in the recent report by the Swedish Government’s Drug Commission of Inquiry [11]. While updated figures on overall HCV prevalence among OAT participants in Stockholm or nationally currently are unavailable, another recent study confirms a significant increase in treatment uptake among PWID in Stockholm, both within and outside of OAT programs. This has led to a significant reduction in HCV prevalence, from 60% in 2017 to 30% in 2021 [46].

The HCV care cascade represents a series of sequential steps in the diagnosis, linkage to care, treatment, reaching SVR and follow-up. However, OAT patients and PWID often face challenges at each step, leading to poor treatment uptake and completion rates. Bringing HCV treatment to the individuals’ geographical locations, including OAT clinics, can significantly improve access and engagement in care, which is also highlighted in the Swedish HCV elimination strategy [31].

Task shifting involves delegating specific responsibilities and tasks to healthcare providers with appropriate training and expertise beyond the traditional roles [47]. Psychiatrists and addiction specialists already play a crucial role in the care of OAT patients and PWID. Expanding the scope of practice for psychiatrists and addiction specialists to include HCV treatment can have several advantages for OAT patients and PWID. This approach could reduce the burden of referrals to specialized clinics, which can be challenging for OAT patients and PWID due to logistical barriers, resulting in missed visits [16]. Bringing HCV treatment to OAT clinics also facilitates a more patient-centered and supportive environment and can reduce the stigma associated with seeking separate specialized care, increasing the likelihood of individuals entering and remaining in HCV care [48]. OAT clinics have often established trusted patient relationships with an understanding of possible specific needs. Thus, incorporating HCV treatment into these practices can provide comprehensive, integrated care that addresses both mental health, substance use disorders and HCV infection.

Expanding the pool of healthcare providers who can prescribe and monitor HCV treatment will result in increased treatment capacity, reduced waiting times, and enhanced accessibility, particularly in areas with limited access to specialized HCV care. Task-shifting to psychiatrists and addiction specialists, will also reduce the workload on ID-specialists, hepatologists and gastroenterologists, allowing a focus on more complex cases and specialized care (e.g., patients with cirrhosis and coinfections), thereby improving the overall efficiency of the healthcare system. However, to implement and maintain effective task-shifting strategies, continuous support and education from HCV specialists is essential and could be advantageously provided through telemedicine [49].

As previously noted, a large proportion of OAT patients are not adequately tested for HCV at OAT clinics. To enhance the HCV treatment cascade and continuum of care, targeted HCV diagnosis efforts, including structured HCV testing and follow-up, are needed. The introduction of psychiatrist-led HCV treatment at the Maria OAT clinic has resulted in a more structured approach to HCV diagnostics, ensuring that all OAT participants are HCV tested upon inclusion and at least annually for those with ongoing risk behaviours, while also providing easy access to HCV treatment. This successful model of care, with psychiatrist-led HCV treatment on-site at OAT clinics, has since 2021 been further implemented at four other OAT clinics in Stockholm. However, streamlining HCV care by utilizing point-of-care testing, introducing patient education, peer-support and simplifying treatment regimens (e.g. introducing pangenotypic treatment) would further enhance treatment accessibility and completion rates among OAT patients and PWID [18, 50, 51].

During the COVID-19 pandemic, the number of HCV treatments decreased worldwide and in Sweden [25, 52]. Between 2019 and 2020, HCV treatment initiations at ID clinics decreased by over 50% and remained at that level during 2021–2022 (personal communication with registry holder for InfCare Hepatitis). Experiences have varied by region, with e.g., disrupted ID outreach activities to OAT clinics to full or temporary pauses in treatment starts at ID clinics that instead needed to focus on COVID-19 [25]. HCV treatments were also discontinued or decreased at many NSP as these programs are mainly managed by ID clinics in Sweden. At the Maria OAT clinic, there was no discontinuation of HCV treatment on-site since psychiatrists themselves treated HCV. However, a 42% decrease in HCV starts in 2020 compared to 2019 was noted, although treatment numbers increased again in 2021. HCV treatment education for psychiatrists, addiction specialists and staff at OAT clinics thus makes HCV treatment and care more sustainable for OAT patients, than e.g., relying on referrals, intermittently funded projects or that only ID specialists treat HCV, which was specifically noted during the COVID-19 pandemic.

Some limitations of our study need to be addressed. The outcomes were based on real-world, convenience sampling data from HCV-treated participants on-site at the Maria OAT clinic and may thus not be generalizable to other treatment settings. Additionally, our study design lacked a control group, which means that our model of care cannot be fully evaluated, as the HCV-treated participants eventually could have received HCV treatment elsewhere. Most HCV-treated participants were found in the high treatment needs group, and OAT patients in the moderate- and low treatment needs groups might have received HCV treatment elsewhere, which was not investigated in this study. As an effect, possible unknown treatments and reinfections in those groups could affect overall SVR and reinfection rates in the whole Maria OAT cohort, most likely resulting in overall higher SVR rates and lower reinfection rates, as there is a suggested lower levels of high-risk drug use in the moderate- and low treatment needs groups. On the other hand, a strength of our study was that HCV treatments in the high treatment needs group were all performed on-site at the OAT clinic and not elsewhere, indicating valid treatment outcomes in this group and a successful model of care. Still, some OAT participants may have missed HCV investigation and treatment due to lack of engagement and other reasons not investigated in this study. A further limitation is that we lack data on individual risk behavior and IDU post SVR and instead rely on a general perception of risk behavior in the differentiated OAT treatment groups. However, most reinfections were expectedly noted in the high treatment needs group, where continuous IDU was highly prevalent. Lastly, we performed no phylogenetic testing or genotype testing on those with viral recurrence between EOT and SVR. Thus, we cannot fully differentiate virological treatment failure from reinfection. However, all participants with viral recurrence were HCV RNA negative at EOT which indicates a high possibility for cure, rather than virological failure. This may have led to an underestimation of reinfections in this study.

## Conclusions

Introducing psychiatrist-led HCV treatment at an OAT clinic was effective, with good treatment results and levels of reinfections consistent with the literature. Enhancing the HCV care cascade and continuum of care for OAT patients offer significant benefits and is essential for local, and global HCV elimination. Task shifting involving psychiatrists and addiction specialists in the treatment of HCV not only ensures integrated care but also optimizes healthcare resources. These approaches, coupled with comprehensive harm reduction strategies, can effectively address the challenges associated with HCV among OAT patients and PWID, leading to improved treatment uptake, completion rates, and ultimately, better health outcomes.

## Data Availability

The datasets used in the study are available from the corresponding author on reasonable request.

## Abbreviations

APRI: Aspartate Aminotransferase (AST) to Platelet Ratio Index
CI: Confidence Interval
COVID-19: Coronavirus disease 2019
ID: Infectious diseases
EOT: End of treatment
DAA: Direct acting antiviral
HCV: Hepatitis C virus
HTN: High treatment needs
LTFU: Lost to follow-up
LTN: Low treatment needs
LSM: Liver stiffness measurement
MTN: Moderate treatment needs
NSP: Needle and syringe programs
OAT: Opioid agonist treatment
PWID: People who inject drugs
PY: Person-years
RNA: Ribonucleic acid
SVR: Sustained virological response
WHO: World Health Organization
